# Arthritis or an Adjacent Fascial Response? A Case Report of Combined Pyomyositis and Aseptic Arthritis

**DOI:** 10.1155/2024/2608144

**Published:** 2024-06-25

**Authors:** Noa Martonovich, Sharon Reisfeld, Yaniv Yonai, Eyal Behrbalk

**Affiliations:** ^1^ Orthopedic Surgery Department Hillel Yaffe Medical Centre, Hadera, Israel; ^2^ Rappaport Faculty of Medicine Technion, Haifa, Israel; ^3^ Infectious Diseases Unit Hillel Yaffe Medical Centre, Hadera, Israel

## Abstract

Pyomyositis, accompanied by aseptic arthritis, has been previously documented in several publications. However, none of the authors in the mentioned case reports offered a pathophysiological explanation for this unusual phenomenon or proposed a treatment protocol. We present a case of a healthy, 70-year-old male who was presented to the emergency department 4 days after tripping over a pile of wooden planks and getting stabbed by a nail to his thigh. The right thigh was swollen. Unproportional pain was produced by a light touch to the thigh. A laboratory test and a CT scan were obtained. The working diagnosis was pyomyositis of the thigh and septic arthritis of the ipsilateral knee. The patient underwent urgent debridement and irrigation of his right thigh. An arthroscopic knee lavage was performed as well. Intraoperative cultures from the thigh revealed the growth of *Streptococcus pyogenes* and *Staphylococcus aureus*. Cultures from synovial fluid were sterile; thus, septic arthritis was very unlikely. The source of the knee effusion might have been an aseptic inflammatory response due to the proximity of the thigh infection. Anatomically, the quadriceps muscle inserts on the patella, and its tendon fuses with the knee capsule, creating a direct fascial track from the thigh to the knee. The inflammatory response surrounding the infection may have followed this track, creating a domino effect, affecting adjacent capillaries within the joint capsule, and causing plasma leakage into the synovial space, leading to joint effusion. Our suggested treatment is addressing the primary infection with antibiotics and considering adding anti-inflammatory therapy, given our suspicion that this process has an inflammatory component.

## 1. Introduction

Pyomyositis is an acute bacterial infection of skeletal muscles, with or without abscess formation. It was first described in 1885 in Japan [1, 2] and was previously known as tropical pyomyositis, earning its name from early 20th-century studies that showed a significantly higher incidence in tropical climates, attributed to the consistently hot and humid weather [3]. However, in the 21st century, similar cases are increasingly being reported in temperate countries [2, 4]. Ngor et al. [5], in a recent systematic review, found that half of the case series originated from high-income temperate countries. Other studies found an increase in pyomyositis incidence in the USA [6, 7] and Australia [8].

Some authors suggest that in tropical countries, it mainly affects young and healthy individuals, while in temperate countries, it affects immunocompromised adults [6, 9].

Previous studies found a correlation between pyomyositis and other conditions, with diabetes mellitus (DM), HIV, and malignancy having the highest correlation [5, 9–11]. Yu et al. [10], on a single-center retrospective study, found that among patients diagnosed with pyomyositis, 40% suffered from DM, 25% suffered from malignancies, and 10% suffered from autoimmune disease or asthma with long-term steroid usage. According to Ngor et al. [5], individuals with HIV infection were five times more likely to be diagnosed with pyomyositis than those uninfected. Meesiri [12] suggested an association between pyomyositis and systematic lupus erythematosus, while other authors suggested a correlation between hematological malignancies and pyomyositis [13–15]. Most patients show a full recovery with no long-term complications. Complications are often due to late diagnosis and include osteomyelitis, septic arthritis, deep vein thrombosis, and pneumonia [4]. More serious complications include sepsis and septic shock, meningitis, acute respiratory distress syndrome (ARDS), and acute kidney failure (AKI) [4]. The mortality rate is unclear and varies greatly in the literature [5]. One recent publication presented a fatal case of a 6-year-old female with a history of pelvic contusion who developed pyomyositis of the iliopsoas muscle. Due to a delayed diagnosis, the patient deteriorated to septic shock and died approximately 16 hours after admission [16]. Similarly, another recent case report described a 16-year-old, otherwise healthy female who presented with abdominal pyomyositis. A delayed diagnosis of approximately three weeks resulted in her death [17]. The common pathogen is *Staphylococcus aureus*, which occurs in both temperate and tropical areas and has the most common location in the thigh muscles [5, 7, 18–20]. The precise underlying mechanisms of pathophysiology remain unclear. Some authors suggest that the typical mode of dissemination is hematogenic and less frequently via adjacent structures [7, 21, 22]. History may reveal blunt or penetration trauma, exertional exercise, or prolonged vascular insufficiency [6, 9, 23]. However, Bickels et al. [24] reported trauma in less than 5% of cases. Agarwal et al. [9] suggested that trauma to muscle tissue results in iron release from myoglobin. This iron is then used by bacteria to grow and proliferate. Moreover, local hematoma may provide a favorable environment for bacteria to bind. Patients may complain of fever, chills, and myalgia. Physical examination reveals swelling with or without overlying erythema. On palpitation, tenderness with wooden induration is seen [9, 23]. Pyomyositis is sometimes misdiagnosed as cellulitis [25, 26] or may present secondary to cellulitis [27].

Lab tests reveal leucocytosis with a shift to the left. C-reactive protein (CRP) and erythrocyte sedimentation rate (ESR) are usually elevated [6, 9, 23]. Serum muscle enzymes, such as creatine phosphokinase (CPK), tend to remain within normal levels despite muscle destruction [9, 23, 28, 29]. Blood cultures are sterile in 70–90% of patients [9]. When present, the abscess may be aspirated. Verma et al. examined 40 patients and revealed positive cultures in 42.5% of abscess aspirations; among them, 100% showed growth of *Staphylococcus aureus* [30]. In their study, Chattopadhyay et al. investigated 12 patients who underwent abscess aspiration. Their findings indicated that 50% of the cultures were positive for *Staphylococcus aureus*, while 17% of the cultures yielded negative results [18]. Section et al. examined 33 pediatric patients with tissue cultures and found that 50% were positive for *Staphylococcus aureus* and 36% were sterile [19].

On imaging investigation, plain radiographs are usually normal and can be used to rule out other pathologies such as fractures, malignancy, or advanced osteomyelitis [9]. Ultrasound (US) can serve as the first imaging method as it is available, noninvasive, and spares radiation. It demonstrates heterogenic tissue with hypoechoic areas [9] and can differentiate cellulitis from pyomyositis [31]. Point of care US (POCUS) is particularly recommended for children because more advanced imaging techniques often require longer scanning times and sedation [32, 33]. A computed tomography (CT) scan may demonstrate muscle enlargement with heterogeneous attenuation and focal edema [34] although it cannot distinguish between an abscess and a swollen muscle [9]. Magnetic resonance imaging (MRI) is the most sensitive and specific mode of imaging, and it remains the gold standard [6, 7, 9, 21]. In the initial stage of pyomyositis, MRI shows an increased muscle volume and an increased signal on T2-weighted images, along with a loss of normal muscular architecture and variable enhancement on the postcontrast sequences [35, 36]. MRI demonstrates the extent of involvement, the location of fluid collection [6], the presence of an abscess [21], and coexisting pathologies such as cellulitis, osteomyelitis, and arthritis [6, 21, 34]. MRI can further assist in differentiating pyomyositis from necrotizing fasciitis. Pyomyositis is characterized by diffusely hyperintense fluid-sensitive signals and intramuscular abscesses, whereas necrotizing fasciitis presents a peripheral band of hyperintensity. Fascial enhancement occurs in both conditions but is thicker and uneven in pyomyositis [35]. Maravelas et al. [7] demonstrated that nearly 9% of patients exhibited concurrent pyomyositis and septic arthritis. Their findings suggested that joint infection could either lead to secondary pyomyositis or serve as a complication of primary pyomyositis. Gordon et al. [34] demonstrated that among 32 patients, 8 patients had evidence of fluid in the adjacent joint on CT or MRI. The author did not further detail whether the fluid was infected or sterile.

Pyomyositis with aseptic arthritis is a rare condition that was previously described in some case reports [37–43]. Patients present with or without the abovementioned clinical presentation of pyomyositis and an effusion of the proximate joint. The laboratory reveals high inflammatory markers, a positive culture of the muscle tissue, and a negative culture of the joint fluid. Blood culture results can either yield positive or negative findings.

## 2. Case Report

An otherwise healthy 70-year-old male, with a history of penicillin allergy, was presented to the emergency department (ED) with a blunt contusion to his thigh after tripping over a pile of wooden planks. On physical examination, the patient could walk with full weight and without limitations. On inspection, there was no evidence of contusion, soft tissue swelling, hematoma, limb deformation, or open wounds. Ranges of motion of the hip joint, knee, and ankle were normal. He was neurovascularly intact. The only positive sign was that on palpation, there was mild tenderness of the right thigh anteriorly. No fractures, soft tissue swelling, or gas were seen on plain radiographs.

The patient was discharged home with analgesics.

The patient returned to the ED four days later, complaining of severe pain in his right thigh and an inability to move the right hip joint and knee. He also reported a fever with shivering chills in the previous 48 hours. He denied any further injury since his previous presentation; although, after taking a thorough history, he reported a possible penetrating injury to his thigh from a screw presented on one of the planks. On physical examination, the right thigh was swollen, and a pinpoint wound was seen on its lateral aspect without any redness or discharge from the wound. The patient's knee was set in an extended position, and he could not flex it due to severe pain. When inspecting the right knee, no significant warmth, joint swelling, or signs of infection were seen, but unproportional pain and tenderness were produced by a light touch of the thigh and knee. The neurovascular examination revealed no deficit. On laboratory exams: a leucocyte count (WBC) of 28,800 c/uL (normal values: 4,500 to 11,000 c/uL) and a CRP of 330 mg/L (normal values: less than 8 mg/L).

Plain radiographs did not reveal any fractures or abnormalities in the bones.

A CT scan of the thigh was performed before and after an IV iodinated contrast injection.

**Figure 1:**
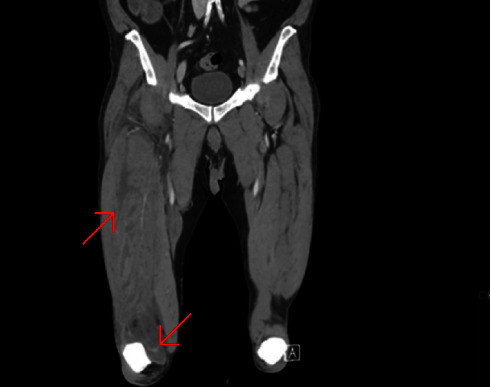
Coronal view of a CT scan, demonstrating an increase in right thigh circumference.

**Figure 2:**
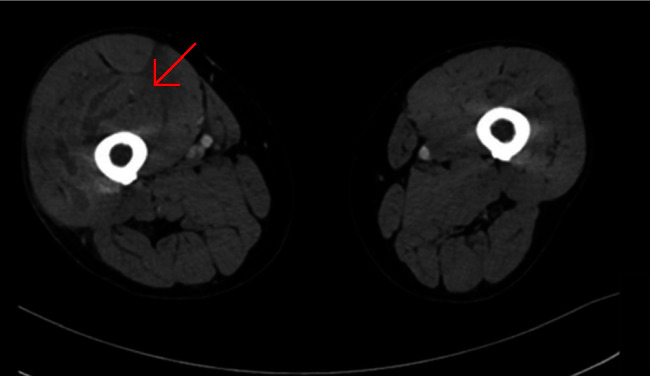
Axial view of a CT scan, demonstrating an increase in right thigh circumference.

The precontrast scan demonstrated a mild hypodensity of the right quadriceps muscle, specifically the vastus intermedius. There was evidence of increased right thigh circumference compared to the left (Figures 1, 2).

On the postcontrast scan, along the vastus intermedius of the quadriceps muscle, there was evidence of fluid dissecting between muscle fibers with a peripheral enhancement. The vastus lateralis was partially involved as well. The regional fascia was involved as well; fluid was seen along the soft tissues of the thigh, up to the iliopsoas muscle. No gas was evident in the soft tissues.

Moreover, the CT scan demonstrated suprapatellar effusion in the right knee (Figure 1).

Based on clinical and radiographic findings, the working diagnosis was thigh muscle pyomyositis or necrotizing fasciitis with knee septic arthritis. As the patient presented with acute, severe systemic sepsis and an inability to move the knee, a decision was made to transfer the patient as soon as possible to the operating room. Therefore, we decided not to aspirate the knee in the ED but rather to perform a complete arthroscopic lavage. Hence, the patient underwent urgent fasciotomy, debridement, and irrigation of his right thigh with an arthroscopic knee lavage.

Arthrocentesis of his right knee was performed and revealed 15 ccs of clear synovial fluid. Cultures were taken from both the right thigh and the right knee.

The patient was admitted to the orthopedic department and was treated empirically with antibiotics (IV Clindamycin, because of the medical history of an allergic reaction to penicillin) after blood cultures were taken. Due to immobility, prophylactic anticoagulation treatment was initiated. Analgesics were administered as well, with nonsteroidal anti-inflammatory drugs (NSAIDs) included. Gram stain of the synovial fluid was negative, WBC from the knee was 74,000 c/uL, and glucose was 55 mg/dl (blood glucose was 186 mg/dl).

**Figure 3:**
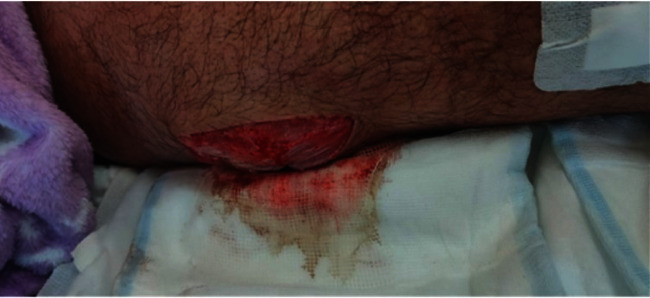
Surgical wound after fasciotomy, debridement, and irrigation of the right thigh. Postoperation day 1.

On postoperation day (POD) 1, blood examination did not demonstrate any significant improvement in acute-phase reactants. The surgical wound on POD1 is shown in Figure 3.

On POD2, the patient underwent a second debridement of the thigh wound and a second arthroscopic knee lavage. The second debridement was performed to exclude an ongoing deep soft tissue infection (“second look”). The second knee lavage was performed due to the high leukocyte and low glucose count from the first knee lavage to rule out the relatively remote possibility of septic arthritis with a negative synovial fluid culture.

On POD3, blood exams demonstrated a slight improvement in acute-phase reactants (WBC of 24,000 c/uL [PMN 81%] and CRP of 224 mg/L). Moreover, culture results had arrived: thigh wounds cultured with cultivated *Streptococcus pyogenes* and *methicillin-sensitive Staphylococcus aureus* (MSSA). Blood cultures and knee synovial cultures were negative. Later, the treatment was changed to cefazolin and clindamycin to give the best therapy for MSSA as well as group A *Streptococcus*.

The patient was still in pain and could not bear weight or flex his right knee. Physiotherapy treatment was initiated.

On POD 11, after a plastic surgeon consultation, the patient underwent a third superficial tissue debridement and an approximation of wound margins. The debridement of the thigh wound was performed to remove excess fibrin tissue from the wound edges for quicker wound healing.

Due to an improvement in the patient's clinical presentation and laboratory exams, the antibiotic course was stopped, and the patient was discharged home.

The patient reported a significant clinical improvement two weeks after his outpatient clinic visit. On examination, full knee range of motion (ROM) without pain on weight bearing was inspected. On blood exams, inflammatory markers were no longer elevated (WBC was 8.7 c/uL, and CRP was 4.6 mg/L).

## 3. Discussion

We presented a case of pyomyositis of the thigh, with both MSSA and *Streptococcus pyogenes* that grew from the muscle, and a unique presentation of arthritis that was not diagnosed on the first inspection, as it was “masked” by the significant swelling of the ipsilateral thigh. This arthritis was first suspected as septic, but as no organisms grew on aerobic and anaerobic cultures, gram stain negative, and inconclusive synovial fluid chemistry from two different knee lavages, we conclude that the synovial fluid in access is sterile.

*Staphylococcus aureus* was described in the literature as the most common bacterial cause of pyomyositis, mostly in tropical countries [44, 45]. Pyomyositis induced by *Streptococcus pyogenes* was also described in several case reports [46, 47]. It is extremely aggressive, and muscle necrosis may occur [48]. The combination of *Staphylococcus aureus* and *Streptococcus pyogenes* as a cause of pyomyositis was not described previously in the literature, as far as we know. Upon literature review, there were 7 publications presenting a total of 10 cases of pyomyositis with aseptic arthritis [37–43]. These reports included 4 children and 6 adults; among them, 3 suffered from diabetes. Five cases involved the hip joint, three involved the knee, one involved the shoulder, and one involved the elbow. The joint culture was negative in all 10 cases, and the muscle culture was positive for *Staphylococcus aureus* in 7 cases (Table 1). None of the authors of the aforementioned case reports provided a pathophysiological explanation for this unusual phenomenon.

Similar to the abovementioned cases, in our case, septic arthritis was primarily suspected, but a definitive diagnosis could not be established since the synovial fluid was sterile on two different knee arthroscopic lavages. Synovial cultures have a sensitivity of 75–95% [49]. The synovial fluid WBC count of more than 50,000 cells/mcl has high specificity for septic arthritis, while a low glucose level is 85% specific for septic arthritis. However, since our patient presented in this case had a rapid arthroscopic lavage, clear-appearing synovial fluid, and sterile cultures with gram stain negative, we presume that he did not have septic arthritis, although this diagnosis cannot be ruled out.

So why does pyomyositis of the thigh cause a sterile effusion of the adjacent joint?

One can think of two possible pathogenesis:

The first one is reactive arthritis. Reactive arthritis is described as a sterile joint inflammation triggered by an infection that develops days to weeks after the acute infection [50]. Our patient presented with both the onset of myositis and arthritis, so the timeline is inadequate for reactive arthritis.

Furthermore, reactive arthritis usually follows urogenital or gastrointestinal infections [50, 51]. No cases of reactive arthritis following soft tissue infections were found at the time of writing this article. After conducting a literature review and convening a multidisciplinary meeting involving our orthopedic, rheumatology, and infectious disease units to discuss the patient, we concluded that the likelihood of reactive arthritis occurring in our patient was low.

The second possible mechanism is the adjacent fascial response (AFR). Observing the anatomy, the leading hypothesis was an AFR. The intramuscular connective tissue (IMCT) consists of three fascial sheets (endomysium, perimysium, and epimysium) [52].

This IMCT was shown to be continuous with the tendons [52–55]. It was also shown that certain tendons fuse with the adjacent joint capsule [56].

The quadriceps tendon inserts on the patella, and its fibers fuse with the knee capsule [57, 58]. Hence, there is a clear track between the thigh compartments and the knee joint.

During an infection, inflammatory mediators affect vascular permeability, which results in an increase in interstitial fluid [59]. Following the above-mentioned fascial track, this inflammatory response may lead to a domino effect, where inflammation spreads along adjacent structures. Eventually, affecting capillaries within the joint capsule and causing plasma leakage into the synovial space—therefore joint effusion [60]. In other words, the knee effusion might have been a local response due to the fascial continuity of the affected areas.

In the last two decades, the study of fascia has gained significant attention in the anatomical fields, shifting the traditional focus from isolated muscle or capsule structures to the integral roles of connective tissues [61]. Understanding the fascial system provides a more precise comprehension of anatomy, which is essential for diagnosing and treating musculoskeletal issues.

In our case, the fascia structures formed the basis for the AFR hypothesis. We mentioned similar cases involving various muscles and adjacent joints in the upper and lower limbs. The muscle-joint-capsule fascial relationship should be studied specifically for each joint. For instance, in the upper limb, the brachial fascia originates from the intermuscular septum, which provides insertion points for some arm and forearm muscles before merging with the elbow capsule [62].

## 4. Conclusions and Recommendations

Aseptic joint effusion following pyomyositis of an adjacent muscle is described in several case reports. The incidence is unknown.

We presented a case of pyomyositis of the quadriceps and knee effusion of the ipsilateral knee and suggested a hypothesis: There is a myofascial continuity between the muscle and the joint capsule. This continuity creates a track between the muscle and the joint space. Inflammatory mediators due to myositis may trigger adjacent fascial structures along this track, leading to an inflammation reaction in the adjacent joint.

Early diagnosis is challenging, as the initial presentation of AFR arthritis mimics septic arthritis. The authors suggest that, given the nonspecific diagnostic tools currently available, AFR arthritis should be managed as septic arthritis until proven otherwise. Septic arthritis is an orthopedic emergency that requires urgent surgical lavage [63], and delay in treatment can result in joint destruction [64].

Future research is needed to identify rapid and more specific findings to differentiate myositis with septic arthritis from myositis with AFR aseptic arthritis.

Once septic arthritis is ruled out, treatment of AFR should be focused on the main affected site. Infection of the main affected site should be addressed and treated with antibiotics. Given the suspicion that this process has an inflammatory component, incorporating anti-inflammatory therapy may offer potential benefits. Additional therapy should include physiotherapy and analgesics. A further study should be conducted to prove this hypothesis.

## Figures and Tables

**Table 1:** Previously published cases of pyomyositis with aseptic arthritis.

Study	Patient age (years)	Joint involved	Muscle involved	Muscle culture	Joint culture
Acute pyomyositis mimicking septic hip—report of a case Sung et al. [38]	5	Hip	Quadratus femoris	*Staphylococcus aureus*	Sterile

Iliacus pyomyositis mimicking septic arthritis of the hip joint Chen and Wan [39]	8	Hip	Iliacus	*Staphylococcus aureus*	Sterile

Medial thigh abscess mimicking septic arthritis of the knee: A report of two cases Da Assunção et al. [40]	56	Knee	Medial thigh	*Staphylococcus aureus*	Sterile
	36	Knee	Medial thigh	*Staphylococcus aureus*	Sterile

Pyomyositis mistaken for septic hip arthritis in children: The role of MRI in diagnosis and management Shuler et al. [42]	13	Hip	Quadriceps, piriformis	*Staphylococcus aureus*	Sterile
	3	Hip	Gluteal muscles	Negative	Sterile

Pyomyositis presenting as septic arthritis A report of 2 cases Andrew and Czyz [37]	63	Elbow	Wrist extensors	*Staphylococcus aureus*	Sterile
	38	Shoulder	Muscles of the upper arm	*Streptococcus pyogenes*	Sterile

Pyomyositis presenting as septic arthritis Brandy-García et al. [43]	2	Knee	Vastus lateralis	Not mentioned	Sterile

Case report primary MRSA myositis mimicking septic arthritis Abu-Abaa et al. [41]	61	Hip	Gluteus medius, Piriformis	Not mentioned	Sterile

## Data Availability

The data that support the findings of this study are available from both open access and paid sources. Two of the referenced materials are from books and are not freely accessible online. All data generated or analyzed during this study are included in this article, with full bibliographic details provided for both open access and paid sources.
